# The Impact of Nusinersen Treatment on Respiratory Function in Patients with Spinal Muscular Atrophy: A Systematic Review

**DOI:** 10.3390/jcm13216306

**Published:** 2024-10-22

**Authors:** Mona Aldukain, Ali Aldukain, Assal Hobani, Abdulmalik Barakat, Lujain Alsayyed, Maher Alomar, Maha Saad Zain Al-Abeden, Nora Alzoum, Ali Alsuheel Asseri

**Affiliations:** 1 Faculty of Medicine, King Khaled University, Abha 62521, Saudi Arabia; 441800934@kku.edu.sa (M.A.); 439800364@kku.edu.sa (A.A.); 441806699@kku.edu.sa (L.A.); 2 College of Medicine, Ibn Sina National College, Jeddah 22421, Saudi Arabia; 1920127@ibnsina.edu.sa; 3 Faculty of Medicine, King Abdulaziz University, Jeddah 21589, Saudi Arabia; akbarakat@stu.kau.edu.sa; 4 College of Medicine, Sulaiman Al Rajhi University, Al-Bukairiyah 52726, Saudi Arabia; maher.alomar@HMG.LOCAL (M.A.); 191210071@srcolleges.org (M.S.Z.A.-A.); 5 College of Medicine, Princess Nourah bint Abdulrahaman University, Riyadh 11671, Saudi Arabia; 439001498@pnu.edu.sa; 6 Department of Child Health, College of Medicine, King Khalid University, Abha 62529, Saudi Arabia

**Keywords:** muscular atrophy, spinal, nusinersen therapy, respiratory function tests, lung function test, pulmonary function test, peak cough flow

## Abstract

**Background/Objectives**: This systematic review evaluated the impact of nusinersen therapy on respiratory health and function in individuals with spinal muscular atrophy (SMA) and determined whether nusinersen improves pulmonary function, focusing on differences based on patient age and the timing of treatment initiation. **Methods**: A systematic search of PubMed, Ovid Medline, ScienceDirect, and Web of Science databases was conducted up to January 2024 in accordance with the PRISMA guidelines. Thirteen studies were included, comprising clinical trials, observational studies, and case series that focused on respiratory outcomes in SMA patients treated with nusinersen. The data on study design, participant characteristics, nusinersen intervention, respiratory outcomes, and adverse events were extracted. The Joanna Briggs Institute Critical Appraisal Tool was used to assess study quality. A narrative synthesis was conducted to address the heterogeneity of the studies. **Results**: This review found a general trend of improvement in pulmonary function, specifically in forced vital capacity (FVC), although the extent and duration of improvement varied across the studies. Peak cough flow (PCF) and peak expiratory flow (PEF) showed positive trends in some studies, although the results were not consistently significant. Respiratory function improvements were frequently observed, particularly in younger patients and those treated earlier. **Conclusions**: Nusinersen appears to enhance respiratory function and improve motor outcomes in SMA patients, especially with early treatment. However, further research is needed to fully understand its mechanisms and long-term effects on respiratory health in SMA.

## 1. Introduction

Spinal muscular atrophy (SMA) is a rare autosomal recessive neuromuscular disorder affecting approximately 1 in 8000 to 10,000 individuals globally [1]. SMA is characterized by the progressive degeneration of motor neurons, resulting in muscle weakness and atrophy. This condition is caused by mutations in the survival motor neuron 1 (SMN1) gene located on chromosome 5q13 [2,3], resulting in inadequate expression levels of the SMN protein. SMA is the most common cause of child mortality due to genetic disease [4]. Respiratory function in SMA can be severely compromised, resulting in breathing difficulties and a heightened vulnerability to respiratory complications [5]. Managing respiratory aspects is essential for enhancing the overall quality of life in individuals with SMA. The condition presents in various forms, each with its own distinct challenges:

- SMA type 1 (Werdnig–Hoffman disease): This is the most common form, presenting before six months of age. It causes severe symptoms, such as restricted mobility, muscle contractures, skeletal abnormalities, and respiratory problems. Without intervention, most affected children do not survive beyond two years, with respiratory disease being the leading cause of death [6,7,8].
- SMA type 2: This form of SMA manifests between 6 and 18 months. Affected children can sit but are unable to stand or walk without help. Breathing difficulties are common, and life expectancy is shortened, although those affected often reach adolescence or young adulthood [6,7,8].
- SMA type 3 (Kugelberg–Welander disease): This form of SMA manifests after 18 months. Children can walk independently but face mobility challenges. Complications include spinal curvature, contractures, and respiratory infections. With appropriate treatment, people with SMA type III can achieve an average life expectancy [6,7,8].
- SMA type 4: This form of SMA manifests in adulthood after age 21 and causes minimal-to-moderate motor impairment [6,7,8].

Given the critical role of respiratory function in SMA, understanding the respiratory efficacy of nusinersen (Spinraza) is crucial for optimizing treatment strategies and improving outcomes in patients with this debilitating disease. A major advancement in SMA therapeutics was made in 2016 with the approval of nusinersen, also known as Spinraza [9]. It denotes a shift from treating the disease’s symptoms to addressing its underlying cause. Nusinersen, an antisense oligonucleotide, modifies the SMN2 gene’s splicing, increasing the amount of functional SMN protein produced [10,11,12]. This innovative treatment seeks to improve breathing and movement abilities. Particularly in treating spinal muscular atrophy (SMA), the effectiveness of nusinersen has been the subject of significant interest and research in the medical community.

Despite nusinersen’s known benefits for motor function in spinal muscular atrophy (SMA), its impact on respiratory function remains inconsistent and not fully understood. Our systematic review aimed to address this gap by investigating nusinersen’s effects on respiratory outcomes across different SMA types. We compiled data to evaluate nusinersen’s potential as a transformative therapy and sought to provide insights that could guide clinical designs and improve the management of respiratory issues in SMA patients.

## 2. Materials and Methods

We conducted a systematic review in accordance with the Preferred Reporting Items for Systematic Reviews and Meta-Analyses (PRISMA) guidelines (version 5.1.0) [13]. The study protocol was pre-registered in the International Prospective Register of Systematic Reviews (PROSPERO) (ID: CRD42024522371). The guidelines of the *Cochrane Handbook for Systematic Reviews of Interventions* was followed for the conduction of this systematic review [14].

### 2.1. Literature Search and Eligibility Criteria

Our search strategy involved the following electronic databases: PubMed, Ovid Medline, ScienceDirect, and Web of Science. Below is a summary (Table 1) of the search strategies employed for each database. The predefined search terms were (“nusinersen” OR “Spinraza”) AND (“spinal muscular atrophy” OR “SMA”) AND (“respiratory function” OR “forced vital capacity” OR “peak cough flow”). This review included clinical trials, observational studies, and case series focusing on respiratory outcomes associated with nusinersen in patients with SMA without time restriction. On the other hand, non-English studies and review articles were excluded.

### 2.2. Study Selection

After the database search, the studies were imported into the Ryan platform for de-duplication. Three independent reviewers conducted the initial screening by title and abstract. Then, they reviewed the full texts of the selected studies for eligibility. Any disagreements were resolved by consensus. A total of 88 studies were initially identified. After title and abstract screening, 15 studies were selected for full-text review. Finally, 13 studies met all of the inclusion criteria.

### 2.3. Data Extraction

Data extraction was independently carried out by four reviewers using a standardized format to capture the study details (author, year, title, and journal), study design, participant demographics (e.g., gender, age, and SMA type), intervention specifics (e.g., dosage and duration), and the outcomes related to respiratory function (e.g., pulmonary function tests, respiratory muscle strength, and incidence of respiratory complications). Respiratory adverse events associated with nusinersen administration were also documented.

### 2.4. Quality Assessment

The methodological quality of the included studies was assessed using the Joanna Briggs Institute (JBI) Critical Appraisal Tool [15]. Two reviewers independently evaluated each study for potential biases, participant selection, study design, and methodology. Any discrepancies were resolved through consensus between the reviewers or by consulting a third reviewer.

### 2.5. Data Synthesis

Due to the heterogeneity among the included studies, a narrative synthesis approach was employed to evaluate the respiratory efficacy of nusinersen in patients with SMA. This method allowed for a comprehensive and in-depth analysis of the data, taking into account the diverse research designs, outcomes, and patient characteristics reported across the studies.

## 3. Results

### 3.1. Characteristics of Included Studies

**Figure 1:**
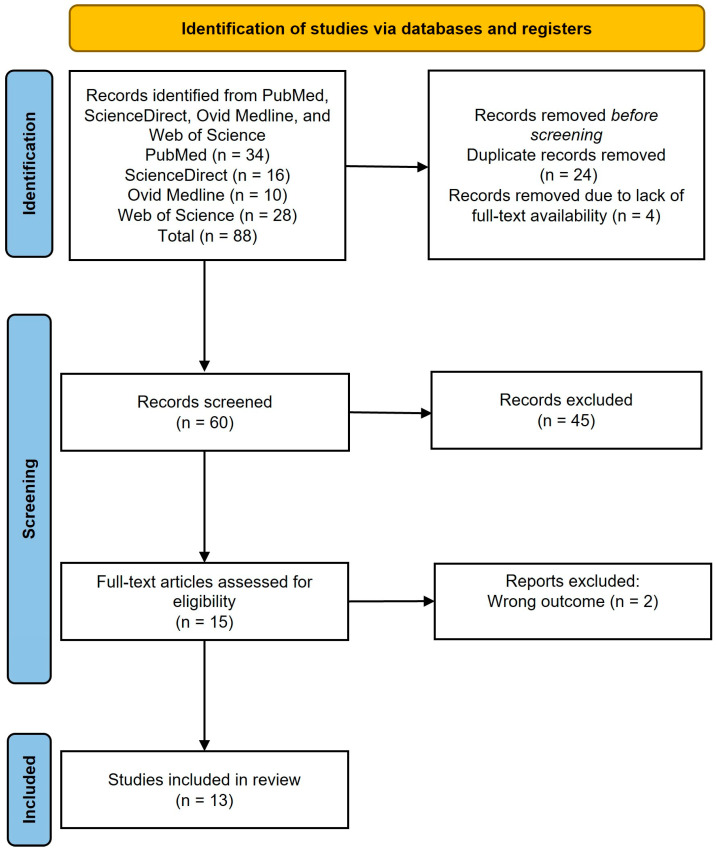
Search and selection process.

A total of 88 studies were initially identified: 34 from PubMed, 16 from ScienceDirect, 10 from Ovid Medline, and 28 from Web of Science. After screening the titles and abstracts, 15 studies were chosen for full-text review. Ultimately, 13 studies fulfilled all of the inclusion criteria (Figure 1) [12,16,17,18,19,20,21,22,23,24,25,26,27], and 2 studies were excluded from full-text screening (Table S1) [28,29].

This systematic review involved 13 papers with 646 participants that reported the pulmonary outcomes of nusinersen administration in SMA patients. Some papers discussed the associations between nusinersen and respiratory and motor functions in various subgroups of SMA patients. For instance, Bjelica et al. established that pulmonary function parameters, including forced vital capacity (FVC), forced expiratory volume 1 (FEV1), and peak expiratory flow (PEF), did not change over treatment durations of up to 30 months; however, PEF was reported to improve among patients using ambulatory services and those admitted with fatigue [16].

On the other hand, the studies conducted by Heitschmidt et al. and Sansone et al. showed mixed evidence related to the effects on respiratory functions [17,18]. Heitschmidt et al. analyzed 12 pediatric patients with SMA types 2 or 3. They found that intrathecal nusinersen treatment did not significantly improve FVC over 300 days, suggesting that nusinersen alone may not enhance respiratory function in these patients [17]. These results emphasize the multidimensional relationships among nusinersen, ambulation, and respiration in pediatric and adult SMA patients with heterogeneous disease progression. Table 2 summarizes the characteristics of the included studies on patients with SMA receiving nusinersen.

### 3.2. Quality Assessment

According to the JBI Critical Appraisal Checklist for Cohort and Experimental Studies, the analyzed studies are of high methodological quality, demonstrating proper study design and implementation. In each study, the study design, selection of participants, exposure, and outcome measurements were appropriately controlled and maintained to be equivalently similar. The follow-up times were stated and considered appropriate, either by presenting complete follow-up data or providing well-elaborated reasons for loss to follow-up. The statistical approaches used in the studies were correct. All of these attributes enhance the totality of internal validity and reliability of the study results. Altogether, the studies are well placed to provide useful data on nusinersen treatment for SMA. By utilizing the research method, they maintain keen adherence to acceptable practices that boost confidence in the discovered outcomes. The quality assessment table (Table S1) provides a structured overview of the methodological strengths and limitations of each study, offering critical insights into the robustness of the evidence concerning nusinersen treatment for spinal muscular atrophy.

The following respiratory-related outcomes emerged from the analysis of these studies.

### 3.3. Changes in Pulmonary Function Tests

Several studies examined the impact of nusinersen on respiratory function, yielding diverse results. Chacko et al. reported stabilization in FVC with nusinersen, reflecting maintained respiratory function over time. Specifically, their study showed a reduction in the annual rate of decline in the FVC z-score among pediatric SMA types 1–3, indicating a slower rate of lung function decline (*p* = 0.02). Additionally, the overall apnea–hypopnea index (AHI) decreased from a median of 5.5 events per hour (IQR 2.1–10.1) at baseline to 2.7 events per hour (IQR 0.7–5.3) after one year [21].

Elsheikh et al. found that nusinersen was safe and well tolerated in adult non-ambulatory SMA patients, with stable FVC and motor function outcomes and increased motor unit and CMAP sizes. However, no significant improvement in functional measures was observed [12]. Similarly, Heitschmidt et al. did not find significant changes in FVC (n = 6; median baseline = 96.0%, 95%-CI [86.5, 110.5]) [17]. In contrast, Bjelica et al. reported no significant changes in the mean FVC or FEV1 from baseline at any time point. However, PEF increased significantly in ambulatory patients at month 30 (+0.8 ± 0.5 L/min) compared to non-ambulatory patients (−0.0 ± 0.5 L/min, *p* < 0.05). Additionally, patients with baseline fatigue showed a significant improvement in the mean PEF at month 10 (+0.6 ± 0.9 L/min) compared to those without baseline fatigue (−0.4 ± 0.5 L/min, *p* < 0.05). The physical domains of the SF-36 positively correlated with changes in FVC and FEV1, while the Fatigue Severity Scale (FSS) negatively correlated with changes in PEF [16].

Furthermore, Walter et al. investigated 19 patients with longstanding 5q-SMA type 3, with 17 completing the 10-month observation period. Although no significant changes were observed in most functional outcome measures, including FVC and FEV1, there were notable improvements in PEF. Specifically, ambulatory patients showed a substantial increase in the mean PEF at day 300 (+0.8 ± 0.5 L/min) compared to non-ambulatory patients (−0.0 ± 0.5 L/min, *p* < 0.05) [19]. Duong et al. observed improvements in specific measures, such as PEF, which showed a significant increase of +0.8 ± 0.5 L/min in ambulatory patients at 30 months (*p* < 0.05). FEV1 and FVC remained stable with no significant changes following nusinersen treatment [20]. Fainmesser et al. reported stability in motor function and a moderate increase in muscular strength over a lengthy observation period, supporting the effectiveness and safety of nusinersen therapy in adult patients with SMA types 2 and 3. However, no significant changes in FEV1 were observed at follow-up visits (*p* = 0.95) [27]. Lastly, Gonski et al. found no significant shift after nusinersen therapy in the predicted FVC%, FVC Z-score, and mean FVC% toward a better state (*p* > 0.05) [25].

### 3.4. Improvement in Respiratory Muscle Strength

Respiratory muscle strength encompasses the capacity of respiratory muscles, including the diaphragm, intercostal muscles, and other inspiratory muscles, to generate forceful and effective respiratory movements. Assessment typically involves tests such as maximal inspiratory pressure (MIP), forced vital capacity (FVC), and esophageal pressure during a maximal sniff, indicative of diaphragm and overall respiratory muscle function. A study by Gómez-García et al. emphasized the significant enhancement in the strength of respiratory muscles among children with SMA type 2. This improvement was evident from better scores in maximal inspiratory pressure, forced vital capacity, and esophageal pressure during sniffing, all showing statistically significant enhancements compared to historical controls, particularly related to improved cough effectiveness and decreased respiratory issues. The strength of the global inspiratory muscles in SMA type 2 patients treated with nusinersen was considerably superior (*p* < 0.05) to the historical controls, as shown in the maximal static inspiratory pressure, forced vital capacity, and esophageal pressure during a maximal sniff [22].

Additionally, Duong et al. analyzed the respiratory function of 42 adults with SMA type 2 or type 3 treated with nusinersen and showed promising trends. The respiratory measures indicated potential improvement, with the most notable change observed in maximum expiratory pressure (MEP), which had a mean annual increase of 6.38 cm H_2_O (95% CI 2.52–10.25). In contrast, maximum inspiratory pressure (MIP) exhibited a mean yearly decrease of −5.50 cm H_2_O (95% CI −11.47 to 0.47), suggesting less consistent improvement. These findings suggest a positive trend in respiratory muscle function, though individual variability was significant, likely due to the heterogeneous sample and varying SMN2 copy numbers among participants [20].

### 3.5. Changes in Ventilatory Support

Regarding changes in ventilatory support, there were mixed results. For instance, Sansone et al. found that most patients with SMA type 1 treated with nusinersen remained stable in their respiratory status over the 10-month follow-up period. Over 80% of the children treated before the age of two years survived, which is significantly higher than the survival rates reported in natural history studies. Among older patients, 75% of those using non-invasive ventilation (NIV) for ≤10 h per day remained stable, as did the majority of those using NIV for >10 h per day or on invasive mechanical ventilation [18]. Similarly, Gonski et al. found that in children with SMA treated with nusinersen, there was a statistically significant improvement in their oxygen nadir during sleep, with the mean increasing from 87.9% to 92.3% (95% CI 1.24–7.63, *p* = 0.01). Additionally, based on clinical and polysomnography (PSG) findings, 6 out of 21 patients (5 with SMA type 2, and 1 with SMA type 3) were able to cease nocturnal non-invasive ventilation (NIV) after starting nusinersen. The authors concluded that while some SMA type 2/3 patients were able to discontinue NIV, there were no significant improvements in the respiratory outcomes overall within two years of starting nusinersen treatment [25].

On the other hand, Pechmann et al. found that during the observation period, the probability of needing ventilator support increased from 29.5% at baseline to 59.7% in the first group and from 38% to 79.0% in the second group at month 38. Logistic regression analysis showed that the SMN2 copy number significantly affected the need for ventilator support. Patients with three SMN2 copies had a lower probability of needing ventilator support under treatment with nusinersen than patients with two SMN2 copies [24]. Additionally, Hepkaya et al. studied 43 patients (18 with type 1, 12 with type 2, and 13 with type 3) with SMA. Follow-up was carried out at the initial dose administration and after four months. In SMA type 1, initially, 13 patients did not require ventilatory support, and 5 patients were tracheostomized. At the 4-month follow-up, seven patients did not require ventilatory support, four were on NIV, and seven were tracheostomized. In SMA type 2, no patients required ventilatory support during follow-up; in SMA type 3, only one patient used NIV for less than 16 h [26].

### 3.6. Timing of Nusinersen Administration

Another determinant that informed the respiratory status was the timing of nusinersen administration, which was evident among symptomatic infants and young children who began treatment early, leading to better respiratory function and fewer respiratory complications in the future. Pechmann et al. noted that early-stage treatment was effective, producing meaningful respiratory gains when patients initiated nusinersen treatment for early-stage SMN deficiency. The progress was more significant in children who began treatment at the age of two years compared to older children. Independence in sitting up was also similarly acquired in 24.5% of children [24]. Similarly, Hepkaya et al. found a significant positive correlation (*p* = 0.026) between beginning nusinersen early and fewer hospital admissions. The group receiving ventilator support observed substantial weight gain and nutritional assistance [26].

However, as noted by Walter et al., the initiation of nusinersen in adults with SMA who had a long duration of the disease provided less respiratory function improvement; it could be inferred that the early initiation of nusinersen could provide better respiratory outcomes [19]. Duong et al. noted improvement trends in patient-reported functional motor and respiratory outcomes, suggesting that nusinersen might be helpful in SMA adults. Forty-two adult SMA participants, whose mean age was 34 years (range of 17–66), were reviewed. They had been taking nusinersen for an average of 12.5 months (3–24 months). Unlike the steady decrease characteristic in untreated individuals, some motor and respiratory assessments indicated improvement [20].

### 3.7. Differences between Pediatric and Adult Patients

This review highlighted that pediatric and adult patients receiving nusinersen exhibited significant disparities in respiratory status. Regarding the differences in the results between pediatric and adult SMA patients, the former, particularly patients with SMA type 1, recorded more significant respiratory gains. Similarly, Chacko et al. and Scheijmans et al. observed considerable improvement in respiratory function among children who received nusinersen; they also reduced the amount of respiratory support required [21,23]. On the other hand, the results obtained for adult subjects were more variable and, often, not so considerable [16,19,27]. These results indicate that although nusinersen benefits pediatric and adult patients, the latter group may benefit from it due to relatively shorter disease duration, milder disease severity, and respiratory differences.

## 4. Discussion

Spinal muscular atrophy (SMA) is a rare and severe genetic disorder characterized by the progressive degeneration of motor neurons, resulting in muscle weakness and significant respiratory complications [5,30,31]. The recent approval of the drug nusinersen (Spinraza) [9,32] represents a considerable advancement in SMA treatment, as it addresses the underlying genetic cause of the disease by modifying the splicing of the SMN2 gene, thereby increasing the production of the functional SMN protein [10,11,12], leading to improved motor and respiratory outcomes [32,33,34,35].

Considering the substantial impact of respiratory involvement on morbidity and mortality among patients with SMA, this study systematically evaluated the impact of nusinersen on respiratory function in SMA patients. The results reveal significant improvements, particularly in pediatric and early-stage cases, and highlight its effectiveness as a transformative therapeutic option. Additionally, recent review articles have comprehensively summarized the available evidence regarding the efficacy and safety of the newly approved therapies for SMA. These treatments include Onasemnogene abeparvovec (Zolgensma^®^, by Novartis, Basel, Switzerland), approved in 2020 with specific eligibility criteria, and Risdiplam (Evrysdi^®^, by Roche, Basel, Switzerland), approved in 2021 and subsequently expanded to include patients of all ages. These advancements represent significant progress in SMA treatment and offer hope for improved outcomes. A detailed comparison of these novel therapies falls outside of the scope of this review [36,37].

### 4.1. Changes in FVC

The results regarding changes in FVC were mixed across the studies. Some studies, such as Chacko et al., found that nusinersen significantly decelerated the decline in the FVC z-score compared to the pre-treatment in their pediatric cohort, indicating improved respiratory function following nusinersen therapy in pediatric SMA types 1–3 [21]. In contrast, Heitschmidt et al. found that FVC did not change significantly in non-ambulatory adults with SMA types 2 and 3 after seven months of treatment [17]. These findings align with broader evidence suggesting that nusinersen provides more substantial respiratory benefits in early-onset disease, while its effects may be more modest in advanced disease [27]. Respiratory muscle strength was another key outcome examined. Fainmesser et al. reported stable respiratory muscle function over 30 months in adults with SMA types 2 and 3 despite improvements in motor function [27]. However, Sansone et al. noted that nusinersen maintained stable respiratory status in children with SMA type 1, though consistent enhancement was not seen across all variables [18]. These mixed results highlight the complex interplay among nusinersen treatment, ambulation, and respiration, which may vary by age and disease stage.

### 4.2. Respiratory Complications

The incidence of respiratory complications was generally low, with infrequent adverse events such as transient respiratory infections or mild discomfort following lumbar puncture [27]. Hepkaya et al. found that early nusinersen treatment reduced hospitalizations and improved enteral feeding in infants with ventilator-dependent SMA type 1 [26]. Pechmann et al. also reported significant motor gains with nusinersen but noted ongoing respiratory support needs, suggesting a more nuanced treatment approach based on disease severity [24].

In summary, nusinersen offers the most significant respiratory benefits in pediatric SMA patients and those in earlier disease stages, enhancing respiratory muscle strength and reducing complications, although its effects in advanced SMA remain variable and require further study. These findings underscore the importance of a personalized, multidisciplinary approach to respiratory management, tailored to age and disease severity, to optimize outcomes and improve the quality of life for individuals with spinal muscular atrophy. Furthermore, managing other comorbid conditions, such as aspiration syndromes, recurrent pneumonias, and sleep-related breathing disorders, is crucial to improve the patient’s overall outcomes.

### 4.3. Implications of the Review

Overall, the findings of the current review have several implications for patients suffering from SMA, independent of its genotype and phenotype, highlighting the importance of conducting well-controlled randomized clinical trials and longitudinal studies to define the effectiveness of this new therapy. Furthermore, the positive effects of early nusinersen initiation emphasize the importance of early diagnosis through newborn screening programs and prompt treatment to optimize respiratory outcomes, particularly among populations with high carrier rates. Additional research is required to elucidate the precise mechanisms through which nusinersen influences respiratory function in SMA patients and to select the best outcome measures that could improve patients’ morbidity and slow disease progression.

### 4.4. Limitations and Future Recommendations

This systematic review synthesized the best available evidence on the relationship between nusinersen therapy and respiratory outcomes in SMA patients across different age groups and subtypes. However, our study has several limitations that need to be considered. First, given the scarcity of well-controlled randomized controlled trials evaluating the efficacy of nusinersen therapy on respiratory outcomes in SMA patients, we conducted a systematic review of observational studies. This approach inherits the potential limitations associated with observational studies, such as confounding factors, selection bias, and limited generalizability. Second, the heterogeneity of the patient populations in these studies, which included both pediatric and adult patients with varying degrees of SMA severity, could potentially limit the generalizability of the findings. Consequently, the present review’s conclusions should be interpreted by taking into consideration these different demographic and outcome factors. Despite challenges such as study heterogeneity and unclear efficacy in long-term disease cases, this review underscores the importance of early intervention and tailored respiratory management. Furthermore, the findings highlight the need for further research on nusinersen’s efficacy in patients with prolonged disease duration and its cost-effectiveness, providing valuable insights for clinicians to optimize treatment and improve respiratory outcomes and quality of life in SMA patients.

## 5. Conclusions

This systematic review found that nusinersen therapy’s impact on respiratory outcomes in SMA patients varied across studies, highlighting both stability and potential improvements in respiratory function. This review also found that nusinersen tended to be more effective in pediatric patients and that early initiation of therapy seems to provide better results. Given the complex relationship between respiratory and motor functions in SMA, further research is needed to clarify nusinersen’s specific effects on respiratory health, emphasizing the importance of comprehensive respiratory monitoring alongside treatment.

## Figures and Tables

**Table 1:** Search strategies for each database.

Database	Search Terms	Search Date
PubMed	(“nusinersen” [Title/Abstract] OR “Spinraza” [Title/Abstract]) AND (“spinal muscular atrophy” [Title/Abstract] OR “SMA” [Title/Abstract]) AND (“respiratory function” [Title/Abstract] OR “forced vital capacity” [Title/Abstract] OR “peak cough flow” [Title/Abstract])	Up to January 2024
Ovid Medline	(“nusinersen” OR “Spinraza”) AND (“spinal muscular atrophy” OR “SMA”) AND (“respiratory function” OR “forced vital capacity” OR “peak cough flow”)	Up to January 2024
ScienceDirect	Title, abstract, keywords: (“nusinersen” OR “Spinraza”) AND (“spinal muscular atrophy” OR “SMA”) AND (“respiratory function” OR “forced vital capacity” OR “peak cough flow”)	Up to January 2024
Web of Science	(AB = (“nusinersen” OR “Spinraza”)) AND AB = (“spinal muscular atrophy” OR “SMA”) AND AB = (“respiratory function” OR “forced vital capacity” OR “peak cough flow”)	Up to January 2024

**Table 2:** Summary of included studies.

Study ID	Study Design	Sample Size	SMA Type	Age Mean (or Median)	Duration of the Intervention (Nusinersen)	Dosage	Main Results
Bjelica et al., 2023 [16]	Observational cohort study	38	SMA types 2 and 3	38.4 years	30 months	NR	Mean FVC, FEV1, and PEF remained stable; for ambulatory patients, mean PEF showed a significant improvement at month 30 (+0.8 ± 0.5 L/min) compared to non-ambulatory patients (−0.0 ± 0.5 L/min), with a *p*-value < 0.05; PEF changes were significantly related to motor function, QoL, and fatigue.
Walter et al., 2019 [19]	Prospective cohort study	17	SMA type 3	Mean age at start of therapy: 35.11 years	300 days	Loading dose of 12 mg at baseline, then maintenance dose every four months	Peak cough flow: significant improvement at visit 5 compared to baseline. No significant changes: apart from peak cough flow, there were no other relevant significant changes in respiratory outcomes at visits 4, 5, or 6 compared to baseline.
Duong et al., 2021 [20]	Prospective cohort study	42	SMA types 2 and 3	34 years	Mean: 12.5 months	Loading dose of 12 mg, maintenance doses every 4 months	Positive changes in motor and respiratory functions; participants stated that they subjectively felt stronger, less fatigued, and had less breathlessness.
Chacko et al., 2021 [21]	Prospective observational study	28	SMA types 1, 2, and 3	Median age: 8.71 years	18 months	Multiple doses were administered over the study period	A reduced FVC rate by −0.25 compared to pre-treatment (−0.58), with a significant difference in decline rates (0.33, 95% CI: 0.02 to 0.66, *p* = 0.04); a better AHI, with significant reduction from a median of 5.5 events/hour to 2.7 events/hour, with a *p*-value of 0.02.
Gomez-García et al., 2021 [22]	Clinical trials using age-matched historical controls	16 participants and 14 historical participants (controls)	SMA types 1c and 2	Mean age: 9.4 ± 2.3 years for participants; mean age of controls: 9.3 ± 1.9 years	14 months	Six injections of nusinersen	Respiratory muscle performance significantly improved in SMA type 2 patients compared to age-matched historical controls. This was assessed through maximal static inspiratory pressure, forced vital capacity (FVC), and esophageal pressure during a maximal sniff, with a *p*-value < 0.05.
Sansone et al., 2020 [18]	Observational, longitudinal cohort study	118	SMA type 1	Median age: 42.8 months	10 months	Intrathecal injections of nusinersen were administered on days 1, 15, 30, and 60 (loading doses) and then every four months (maintenance doses)	More than 80% of children treated before 2 years of age survived without requiring tracheostomy or non-invasive ventilation (NIV) for ≥16 h per day; a somewhat reduced total of NIV hours for children below 2 years of age.
Scheijmans et al., 2022 [23]	Single-center prospective cohort study	71	SMA types 1, 2, and 3	Median age: 54 months	38 months	Treatment started with a loading dose on days 0, 14, 28, and 63, followed by intrathecal injections every four months	Positive change in motor function, with stabilization in 18 percent of patients; no significant changes in respiratory function; 82 adverse effects, with none leading to treatment discontinuation.
Elsheikh et al., 2021 [12]	Prospective observational study	19	SMA types 2 and 3	39.7 ± 13.9 years	Assessed up to 14 months following nusinersen initiation	Participants received intrathecal nusinersen treatment on days 1, 15, 29, and 60, followed by maintenance doses administered every four months	FVC was stable, and functional measures were similar to baseline; CMAP and single motor unit potential amplitudes increased; motor unit counts stabilized.
Pechmann et al., 2023 [24]	Observational, longitudinal cohort study	143	SMA type 1 (with genetic confirmation of 5q SMA)	8.4 ± 6.0 months; cohort 1b: mean age of 89.8 ± 58.4 months	Up to 38 months	NR	These included marked motor function gains, stabilization of respiratory and bulbar involvement, and higher dependency on ventilators and nasal feeds.
Gonski et al., 2023 [25]	Retrospective observational cohort study	48	SMA types 1, 2, and 3	Mean age at first dose: 6.98 years (SD 5.25); SMA 1: mean age at first dose: 0.54 years (SD 0.33); SMA 2: mean age at first dose: 8.90 years (SD 4.96); SMA 3: mean age at first dose: 8.33 years (SD 4.04)	Data collected two years before date of first dose of nusinersen and then for two years after starting nusinersen	NR	Stabilization of respiratory outcomes; no significant changes in lung function and the majority of PSG measurements; significant improvement in oxygen nadir during sleep (mean increased from 87.9% to 92.3%, 95% CI: 1.24 to 7.63, *p* = 0.01); NIV use: 6 out of 21 patients discontinued nocturnal NIV post-treatment.
Hepkaya et al., 2022 [26]	Clinical trial	43	SMA types 1 (n = 18), 2 (n = 12), and 3 (n = 13)	SMA type 1: mean age at diagnosis: 4.39 ± 2.54 months; SMA type 2: mean age at diagnosis: 23.25 ± 21.49 months; SMA type 3: mean age at diagnosis: 64.54 ± 51.23 months	13 months	Intrathecal injections with 12 mg of nusinersen: SMA type 1 (days 1, 15, 29, and 64), SMA types 2 and 3 (days 1, 29, 85, and 274)	Overall, hospitalization was reduced with early intervention, with a *p*-value of 0.026; enteral feeding was better in a ventilator-dependent subgroup.
Fainmesser et al., 2022 [27]	Cohort study	37	SMA types 2 (n = 15) and 3 (n = 22)	38 years	30 months	Intrathecal loading doses of 12 mg nusinersen on days 0, 14, 28, and 63, followed by maintenance doses every 4 months	Modest improvement in motor function up to 6 months, with stabilization thereafter; no significant change in respiratory function assessed by FEV1; only noted side effect was post-lumbar puncture headache.
Heitschmidt L et al., 2021 [17]	Retrospective cohort study	12	SMA types 2 and 3	8.6 years	7 months	NR	FVC did not change significantly by end of study; respiratory function remained essentially unchanged.

SMA: spinal muscular atrophy; NR: not reported; NA: not applicable.

## Supplementary Materials

The following supporting information can be downloaded at: https://www.mdpi.com/article/10.3390/jcm13216306/s1, Supplementary File S1—PRISMA Checklist; Supplementary File S2: Table S1: Reasons for exclusion of studies in full-text screening; Table S2: Quality assessments of the included studies.
