# Supplementary material for: The Impact of Nusinersen Treatment on Respiratory Function in Patients with Spinal Muscular Atrophy: A Systematic Review
Source: J Clin Med. 2024 Oct 22;13(21):6306. doi: 10.3390/jcm13216306 (PMC11546051; doi:10.3390/jcm13216306)
Supplement: Supplementary file 1 [file jcm-13-06306-s001.zip › Supplementary file S2.pdf]

Table S1. Reasons for exclusion of studies in full-text screening

Excluded study	Reason for exclusion
L. Edela et al. [28]	Not align with the specific outcome of interest, focuses on evaluating and developing a respiratory scoring system rather than providing direct evidence or detailed analysis on the effects of nusinersen on respiratory function in SMA patients.
E. Salort-Campana et al. [29]	Not align with the specific outcome of interest, discusses the role of multidisciplinary team meetings in decision-making and providing guidelines for SMA treatment in adults. It does not specifically address the direct effects of nusinersen on respiratory function.

Table S2. Quality assessments of the included studies

**Cohort study design**									
**Study**	**Were the two groups similar and recruited from the same population**	**Were the exposures measured similarly to assign people to both exposed and unexposed groups?**	**Was the exposure measured in a valid and reliable way?**	**Were confounding factors identified?**	**Were strategies to deal with confounding factors stated?**	**Were the groups/participants free of the outcome at the start of the study (or at the moment of exposure)?**	**Were the outcomes measured in a valid and reliable way?**	**Was the follow-up time reported sufficient to be long enough for outcomes to occur?**	**Was follow-up complete, and if not, were the reasons to loss to follow-up described and explored?**
Bjelica et al., [16]	✗	✗	✓	✗	✗	✓	✓	✓	✓
Walter et al., [19]	✗	✗	✓	✗	✗	✓	✓	✓	✓
Duong et al., [20]	✗	✗	✓	✗	✗	✓	✓	✓	✓

Chacko et al., [21]	✗	✗	✓	✗	✗	✓	✓	✓	✓
Sansone et al., [18]	✗	✗	✓	✗	✗	✓	✓	✓	✓
Scheijmans et al. [23]	✗	✗	✓	✗	✗	✓	✓	✓	✓
Elsheikh et al. [12]	✗	✗	✓	✗	✗	✓	✓	✓	✓
Pechmann et al. [24]	✗	✗	✓	✓	✗	✓	✓	✓	✓
Gonski et al., [25]	✗	✗	✓	✗	✗	✓	✓	✓	✓
Fainmesser et al., [27]	✗	✗	✓	✗	✗	✗	✓	✓	✓
Heitschmidt et al., [17]	✗	✗	✓	✗	✗	✓	✓	✓	✓
**Experimental study design**									
**Study**	**Is the study clear about what the 'cause' and what the 'effect' are?**	**Were the participants included in any comparisons similar?**	**Were the participants included in any comparisons receiving similar treatment/ care, other than the exposure or intervention of interest?**	**Was there a control group?**	**Were there multiple measurements of the outcome both pre and post the intervention/exposure?**	**Was follow up complete and if not, were differences between groups in terms of their follow up adequately described and analyzed?**	**Were the outcomes of participants included in any comparisons measured in the same way?**	**Were outcomes measured in a reliable way?**	**Was appropriate statistical analysis used?**
Gómez-García et al., [22]	✓	✗	✗	✓	✗	~	~	✓	✓
Hepkaya et al., [26]	✓	✓	✓	✗	✓	✓	✓	✓	✓

✓, low risk of bias; ✗, high risk of bias; ~, and moderate risk of bias/some concerns
